# Association Between Stress Management Interventions and Symptom Severity in Adults With Irritable Bowel Syndrome (IBS) in Saudi Arabia

**DOI:** 10.7759/cureus.102466

**Published:** 2026-01-28

**Authors:** Randa M Alharazi, Intessar Sultan, Gharam A Alahmadi, Renad Ghandour, Mohamad B Dahha, Yousef T Rajikhan, Emad Al Takroni, Layan Alahmadi, Shatha Almahwzi, Salman alhrbi

**Affiliations:** 1 Internal Medicine, Ibn Sina National College for Medical Studies, Jeddah, SAU; 2 Medicine, Ibn Sina National College for Medical Studies, Jeddah, SAU; 3 Medicine and Surgery, Ibn Sina National College for Medical Studies, Jeddah, SAU; 4 Medicine, Taibah University, Madinah, SAU

**Keywords:** ibs, irritable bowel syndrome, rome iv criteria, stress management, symptom severity

## Abstract

Background: Psychological stress is a major contributing factor to irritable bowel syndrome (IBS), a widespread functional gastrointestinal disorder. Consequently, understanding the relationship between stress management strategies and the severity of IBS symptoms is crucial.

Aim: This study sought to assess the association between stress management interventions and symptom severity among adults with IBS in Saudi Arabia. Furthermore, it investigated the associations between IBS, demographic data, lifestyle habits, and perceived stress levels.

Methodology: We conducted a cross-sectional study involving adults aged 18 and older in Saudi Arabia who had a clinical diagnosis of IBS. Participants completed a validated, bilingual web-based survey that incorporated the Rome IV diagnostic criteria, the IBS Symptom Severity Scale (IBS-SSS), and the Perceived Stress Scale (PSS). Statistical significance for both descriptive and inferential analyses was established at p < 0.05.

Results: The study included 517 participants, of whom 24% utilized stress management techniques. The most prevalent methods were deep breathing (66.9%), meditation (50.8%), and yoga (30.6%). Comparative analysis showed significantly lower symptom severity scores among those practicing stress management, specifically regarding the frequency of abdominal pain (p = 0.010), abdominal distension (p < 0.002), satisfaction with bowel habits (p < 0.001), and interference with daily activities (p = 0.007). Analysis indicated that individuals engaging in stress management reported fewer severe symptoms and lower perceived stress, whereas high stress levels were significantly more common among non-practitioners (p = 0.007). Gender and lifestyle choices were also significantly linked to IBS burden.

Conclusion: The use of stress management protocols is associated with reduced symptom severity and lower perceived stress in IBS patients. These results advocate for the integration of stress reduction techniques into comprehensive IBS care plans.

## Introduction

The status of one's gastrointestinal system is pivotal to overall well-being, influencing both physical and mental health. A functioning digestive tract is required for the absorption of nutrients essential for cellular repair, energy, and growth. However, common disorders such as constipation, bloating, and irritable bowel syndrome (IBS) often disrupt daily functioning, causing anxiety, discomfort, and a diminished quality of life [1].

IBS is defined clinically by chronic abdominal pain and irregular bowel habits, affecting a large demographic. Typical presentations include bloating and constipation, which can further aggravate emotional distress [2]. For patients with IBS, regulating stress is critical for improving both physical and mental health outcomes. Stress triggers the gut-brain axis, resulting in symptoms such as altered bowel motility and abdominal pain. Furthermore, stress-induced hormonal fluctuations can heighten gut sensitivity, thereby complicating the clinical picture [3].

Adopting stress management strategies offers substantial advantages for individuals with IBS, including symptom alleviation, enhanced coping mechanisms, and improved quality of life. Effectively addressing stress allows individuals to better manage gastrointestinal issues. Globally, the importance of stress management in healthcare is increasingly recognized. Many nations are adopting mental wellness programs, integrating techniques like mindfulness and yoga into medical care. Consequently, organizations are developing wellness initiatives to help personnel manage stress, which ultimately boosts productivity and general health [4].

In the context of Saudi Arabia, stress management is particularly relevant due to specific cultural and lifestyle dynamics. Rapid modernization has introduced new stressors impacting public health. However, traditional practices, such as prayer and meditation, can be effectively incorporated into stress reduction strategies. Additionally, social support through family and community ties remains essential. Given the pressures of education and employment, effective stress management is vital for improving life quality in this region [5].

This study aimed to evaluate the effect of stress management interventions on symptom severity in adults with IBS in Saudi Arabia. It further examined the relationship between stress levels and IBS symptoms, explored the influence of lifestyle and dietary factors, and assessed changes in symptom severity before and after intervention.

## Materials and methods

Study design

We utilized a cross-sectional, questionnaire-based design. A self-administered online survey was designed to evaluate the relationship between stress management interventions and symptom severity among adults diagnosed with IBS in Saudi Arabia.

Sampling and population

A non-probability convenience sampling approach was employed to recruit participants from the general public. The survey link was disseminated electronically via major social media platforms to ensure a broad reach. The cohort comprised male and female residents of Saudi Arabia, aged 18 years and older, with a clinical diagnosis of IBS. The sample represented diverse demographic characteristics, dietary patterns, and gastrointestinal symptom profiles.

Data collection

Following a comprehensive literature review and expert consultation, a structured bilingual questionnaire (Arabic and English) was created using Google Forms (Google, Mountain View, CA, USA). The instrument comprised the following sections: (1) informed consent form; (2) demographic information; (3) lifestyle and dietary habits; (4) Rome IV diagnostic criteria for IBS [6]; (5) IBS Symptom Severity Scale (IBS-SSS) [7] (permission to use this scale was obtained from the copyright holder via the Copyright Clearance Center); (6) Perceived Stress Scale (PSS) [8] (permission for its use was obtained in accordance with the author’s requirements); and (7) details of stress management interventions.

For the purpose of this study, a "healthy diet" was defined as the self-reported regular consumption of nutrient-dense foods (such as fruits, vegetables, and whole grains) and the limitation of processed foods, consistent with general dietary guidelines.

Instrument validation and reliability

Subject matter experts validated the questionnaire content. A pilot study involving 10% of the calculated sample size was conducted to ensure comprehension, clarity, and feasibility. Cronbach’s alpha was utilized to evaluate internal consistency and reliability.

Sample size calculation

The sample size was calculated using the Cochran formula (n = z^2^pq/d^2^) based on an estimated IBS prevalence of 17% in Saudi Arabia, a 95% confidence level, and a 5% margin of error. This calculation indicated a minimum required sample size of 217 participants, which was exceeded by our final sample of 517.

Ethical considerations

The study adhered to ethical standards and guidelines. Ethical approval was obtained from the Institutional Research Review Board (IRRB) of Ibn Sina National College for Medical Studies (No: IRRB-01-07092025). Participation was voluntary, with informed consent obtained electronically. No personal identifiers were collected, and data were stored anonymously and securely for research purposes.

Statistical analysis

Data was coded, cleaned, and analyzed using IBM SPSS Statistics for Windows, Version 23.0 (Released 2015; IBM Corp., Armonk, NY, USA). Descriptive statistics summarized variables, with categorical data presented as frequencies and percentages, and continuous variables as means ± standard deviations (SD). To examine associations between stress management and IBS symptom severity, chi-square or Fisher’s exact tests were used for categorical variables, independent t-tests or ANOVA were used for continuous variables, and a p-value < 0.05 was considered statistically significant.

## Results

Demographic and lifestyle factors

A total of 517 adults diagnosed with IBS were included, of whom 124 (24%) practiced stress management. The cohort was predominantly female (63.1%); however, the proportion of females was significantly higher in the non-stress management group compared to the stress management group (71.1% vs. 37.4%, respectively; p < 0.001). The median age was significantly lower in the stress management group (23 years, IQR 9) compared to the non-management group (27 years, IQR 17; p = 0.005). Education level, occupation, and duration of IBS did not differ significantly between the groups. Participants practicing stress management showed significantly higher engagement in healthy dietary and exercise habits compared to non-practitioners (p = 0.004 and p < 0.001, respectively). Smoking status did not differ significantly (p = 0.630). Notably, perceived stress was significantly lower in the stress management group, with fewer participants reporting high stress levels (3.3%) compared to the non-management group (13.5%; p = 0.007) (Table 1).

**Table 1:** Characteristics of IBS patients who received and did not receive stress management Data are presented as numbers and percentages. p-values were calculated using the Pearson chi-square test (χ^2^). Significant associations were found for gender (χ​​​​​​​^2 ^= 30.01, df = 1), dietary habits (χ​​​​​​​^2 ^= 10.99, df = 2), exercise (χ​​​​​​​^2 ^= 23.36, df = 4), and perceived stress (χ​​​​​​​^2 ^= 9.87, df = 2). *Statistically significant difference (p < 0.05).

Characteristics		Participants (N = 517; 100%)		No stress management (n = 393; 76%)		Received stress management (n = 124; 24%)		p-value
		Number	Percentage	Number	Percentage	Number	Percentage	
Gender	Female	326	63.1	280	71.1	46	37.4	<0.001*
	Male	191	36.9	114	28.9	77	62.6	
Age, years (median, IQR)		26 (13)		27.0 (17)		23.0 (9)		0.005*
Education	Post-graduate studies	32	6.2	20	5.1	12	9.8	0.124
	Bachelor's degree	323	62.5	246	62.4	77	62.6	
	High school	135	26.1	104	26.4	31	25.2	
	< High school	27	5.2	24	6.1	3	2.4	
Occupation	Employed	206	39.8	151	38.3	55	44.7	0.136
	Student	189	36.6	142	36.0	47	38.2	
	Unemployed	122	23.6	101	25.6	21	17.1	
Duration of IBS	<1 year	229	44.3	120	30.5	42	34.1	0.292
	1-3 years	162	31.3	182	46.2	47	38.2	
	>3 years	126	24.4	92	23.4	34	27.6	
Dietary habits	Healthy	50	9.7	32	8.1	18	14.6	0.004*
	Moderately healthy	331	64.0	246	62.4	85	69.1	
	Unhealthy	136	26.3	116	29.4	20	16.3	
Exercise	Daily	51	9.9	33	8.4	18	14.6	<0.001*
	Several times a week	132	25.5	90	22.8	42	34.1	
	Once a week	134	25.9	97	24.6	37	30.1	
	Rarely	157	30.4	133	33.8	24	19.5	
	No	43	8.3	41	10.4	2	1.6	
Smoking	No	448	86.7	343	87.1	105	85.4	0.630
	Yes	69	13.3	51	12.9	18	14.6%	
Perceived stress	Low stress	20	3.9	15	3.8	5	4.1	0.007*
	Moderate stress	440	85.1	326	82.7	114	92.7	
	High stress	57	11.0%	53	13.5	4	3.3	

Characteristics of Stress Management

Among the 124 participants utilizing stress management, the most common methods were deep breathing (66.9%) and meditation (50.8%), followed by yoga (30.6%) and cognitive behavioral therapy (23.4%). Over half of the participants (53.7%) used a single method, while 8.1% combined all four methods. Practice frequency varied, with "several sessions a week" being the most common (35.0%), followed by once a week (29.3%), rarely (19.5%), and daily sessions (16.3%) (Table 2).

**Table 2:** Characteristics of stress management received by IBS patients

Stress management variables		Participants (n = 124)	Percentage
Types of stress management	Deep breathing	83	66.9
	Meditation	63	50.8
	Yoga	38	30.6
	Cognitive behavioral therapy	29	23.4
Number of stress management types	1	66	53.7
	2	34	27.6
	3	13	10.6
	4	10	8.1
Frequency of stress management sessions	Rarely	24	19.5
	Once a week	36	29.3
	Several times a week	43	35.0
	Daily	20	16.3

Comparison of IBS symptoms before and after stress management

**Figure 1:**
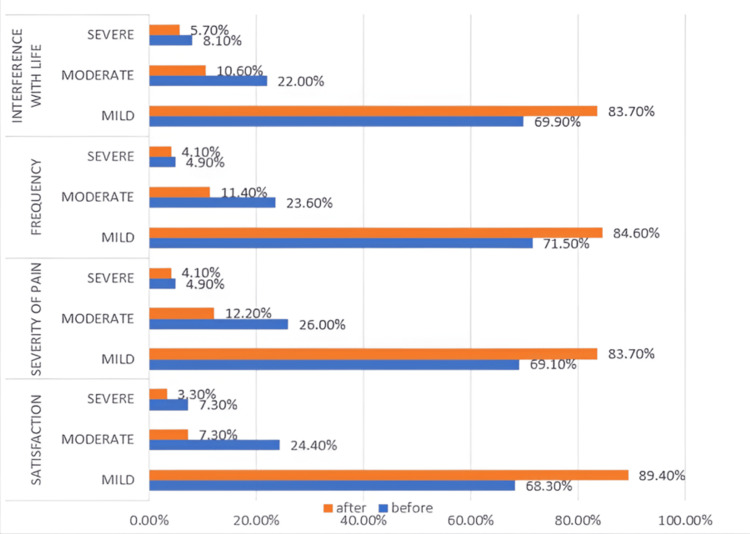
Significant differences in IBS symptom severity before and after stress management

Significant improvements were noted after adopting stress management. The frequency of abdominal pain declined, with 84.6% reporting mild pain post-intervention compared to 71.5% beforehand (p = 0.010). Abdominal distention improved, with 83.7% reporting mild distention compared to 69.1% previously (p < 0.002). Satisfaction with bowel habits rose significantly, with 89.4% reporting mild dissatisfaction post-intervention compared to 68.3% before (p < 0.001). Interference with daily life also decreased, with 83.7% reporting mild impact compared to 69.9% before (p = 0.007). While the severity of abdominal pain decreased (81.3% mild severity post-intervention vs. 71.5% before), this change was not statistically significant (p = 0.141) (Figure 1).

Comparison of IBS symptoms between participants with and without stress management

Compared to non-practitioners, those who practiced stress management reported significantly less severe abdominal pain (3.3% vs. 15.2%; p < 0.001), less frequent abdominal pain (4.9% vs. 13.2%; p = 0.001), milder abdominal distention (69.1% vs. 61.7%; p = 0.001), less interference with daily life (69.9% vs. 59.6%; p < 0.001), and lower perceived stress (3.3% vs. 13.5%; p = 0.007). No significant difference was observed regarding satisfaction with bowel habits (p = 0.060) (Table 3).

**Table 3:** Comparison between IBS participants who received and who did not receive stress management Data are presented as numbers and percentages. p-values were calculated using the Pearson chi-square test (χ^2^). Significant differences were observed for abdominal distension frequency (χ^2 ^= 10.87, df = 4), change in bowel habits (χ^2 ^= 15.34, df = 4), pain severity (χ​​​​​​​^2 ^= 15.22, df = 2), pain frequency (χ​​​​​​​^2 ^= 14.28, df = 2), distension severity (χ​​​​​​​^2 ^= 13.91, df = 2), interference with life (χ​​​​​​​^2 ^= 20.35, df = 2), and perceived stress (χ​​​​​​​^2 ^= 9.87, df = 2). *Statistically significant difference (p < 0.05).

IBS symptoms		Stress management				p-value
		Without stress management		With stress management		
		n	%	n	%	
Abdominal pain	No	73	18.5	20	16.3	0.119
	Rarely	126	32.0	40	32.5	
	Sometimes	104	26.4	45	36.6	
	Often	62	15.7	14	11.4	
	Very often	29	7.4	4	3.3	
Distension	No	78	19.8	21	17.1	0.028*
	Rarely	129	32.7	40	32.5	
	Sometimes	97	24.6	44	35.8	
	Often	58	14.7	16	13.0	
	Very often	32	8.1	2	1.6	
Change in bowel habits	No	92	23.4	30	24.4	0.004*
	Rarely	125	31.7	42	34.1	
	Sometimes	98	24.9	42	34.1	
	Often	43	10.9	9	7.3	
	Very often	36	9.1	0	0.0	
Severity of pain	Mild	244	61.9	88	71.5	<0.001*
	Moderate	90	22.8	31	25.2	
	Severe	60	15.2	4	3.3	
Frequency	Mild	248	62.9	88	71.5	0.001*
	Moderate	94	23.9	29	23.6	
	Severe	52	13.2	6	4.9	
Distension severity	Mild	243	61.7	85	69.1	0.001*
	Moderate	100	25.4	32	26.0	
	Severe	51	12.9	6	4.9	
Satisfaction with bowel habits	Mild	251	63.7	84	68.3	0.060
	Moderate	96	24.4	30	24.4	
	Severe	47	11.9	9	7.3	
Interference with life	Mild	235	59.6	86	69.9	<0.001*
	Moderate	91	23.1	27	22.0	
	Severe	68	17.3	10	8.1	
Perceived stress	Low	15	3.8	5	4.1	0.007*
	Moderate	326	82.7	114	92.7	
	High	53	13.5	4	3.3	

## Discussion

This research sought to assess the value of stress management in alleviating IBS symptoms among adults in Saudi Arabia. Regarding demographics, our data indicates a female predominance among IBS patients, a finding consistent with existing literature [9,10]. Conversely, stress management techniques were utilized most frequently by younger individuals and males.

Significant differences in lifestyle behaviors were noted between those who practiced stress management and those who did not. The stress management group was more inclined toward regular physical activity and healthier dietary habits. These behaviors are known to modulate IBS symptoms and may work synergistically with stress reduction to enhance outcomes. Consistent with the systematic review by Sirri et al., our results showed no significant correlation between smoking status and the severity of IBS symptoms [11].

Analysis of perceived stress revealed a distinct pattern: while moderate stress was common across the board, high stress levels were significantly more prevalent in the non-intervention group. This corroborates prior research identifying perceived stress as a core component of IBS pathophysiology, where elevated stress exacerbates visceral hypersensitivity and disrupts gut-brain signaling [12,13]. Our data support previous assertions that high stress predicts poorer IBS prognoses [14].

Interventions such as cognitive behavioral therapy (CBT), mindfulness, and relaxation training have been proven to lower perceived stress and mitigate IBS severity [15,16]. This underscores the dual role of stress management in providing symptom relief and reducing the psychological burden of the disorder.

Furthermore, participants engaging in stress management reported significant reductions in abdominal distension, pain frequency, and interference with daily life, alongside higher satisfaction with bowel habits. Yoga, meditation, and deep breathing were the primary methods used, typically practiced several times weekly. These results align with previous studies linking CBT and mindfulness-based interventions to improved health-related quality of life and reduced symptom severity [16-18]. Consequently, stress reduction should be prioritized as a non-pharmacological strategy in IBS care, especially for patients with psychological symptoms or in resource-constrained environments.

Strengths and limitations

This study's strengths include a relatively large sample size and its focus on the Saudi Arabian population, an underrepresented demographic in IBS research. However, limitations exist. The cross-sectional design restricts causal inference. Reliance on self-reported measures may introduce recall and social desirability biases. Additionally, the assessment of stress management was limited to binary values without accounting for the quality, duration, or specific type of intervention.

## Conclusions

This study examined the association between stress management interventions and symptom severity among adults with IBS in Saudi Arabia. Significant associations were found between gender, lifestyle factors, perceived stress, and IBS symptom burden. Stress management practices were common, with deep breathing, meditation, and yoga being the most frequently used. Symptom severity, including abdominal pain, distention, bowel habit dissatisfaction, and interference with daily life, was significantly lower among participants practicing stress management. Moreover, participants who utilized stress management reported lower rates of severe symptoms compared to those who did not. These findings highlight the importance of integrating stress reduction strategies into the holistic management of IBS.

## Appendices

**Table 4:** Demographic and lifestyle questionnaire

Item no.	Question	Response options
1	Do you agree to participate in this study?	Yes/No
2	Researcher ID	Numeric
3	Are you a resident of Saudi Arabia?	Yes/No
4	Have you been diagnosed with irritable bowel syndrome (IBS)?	Yes/No
5	Gender	Male/Female
6	Age	Years
7	Academic degree	Less than high school/High school/Bachelor’s degree/Postgraduate
8	Occupation	Student/Employed/Unemployed
9	Duration since IBS diagnosis	<1 year/1-3 years/>3 years
10	How would you describe your diet?	Healthy/Moderately healthy/Unhealthy
11	How often do you exercise?	Daily/Several times a week/Once a week/Rarely/Never
12	Do you smoke?	Yes / No
13	Do you consume alcohol?	Yes / No
14	Additional comments (optional)	Free text

**Table 5:** Stress Management Practices Questionnaire

Item No.	Question	Response Options
1	Have you ever participated in any stress management programs?	Yes / No
2	How often do you practice stress management techniques?	Daily / Several times a week / Once a week / Rarely / Never
3	What types of stress management techniques do you use?	Meditation / Deep breathing / Yoga / Cognitive behavioral therapy / Other
4	Severity of IBS symptoms before stress management	0 (none) – 10 (very severe)

**Table 6:** IBS Symptom Severity Scale (IBS-SSS) Source: [7].

Item no.	Item	Scale
1	Severity of abdominal pain	0 (no pain) to 10 (very severe)
2	Frequency of abdominal pain	0 (none) to 10 (very frequent)
3	Severity of abdominal distention	0 (none) to 10 (very severe)
4	Satisfaction with bowel habits	0 (very satisfied) to 10 (very dissatisfied)
5	Interference with life in general	0 (not at all) to 10 (a great deal)

**Table 7:** Perceived Stress Scale (PSS-10) Source: [8].

Item no.	Question	Response options
1	Upset because of something unexpected	0 = never to 4 = very often
2	Unable to control important things in life	0 = never to 4 = very often
3	Felt nervous and stressed	0 = never to 4 = very often
4	Confident about handling personal problems	0 = never to 4 = very often
5	Felt things were going your way	0 = never to 4 = very often
6	Could not cope with all things you had to do	0 = never to 4 = very often
7	Able to control irritations in life	0 = never to 4 = very often
8	Felt on top of things	0 = never to 4 = very often
9	Angered because of things outside your control	0 = never to 4 = very often
10	Felt difficulties piling up too high	0 = never to 4 = very often

## References

[1] Weaver KR, Melkus GD, Henderson WA (2017). Irritable bowel syndrome. Am J Nurs.

[2] Sanders ME, Merenstein DJ, Reid G, Gibson GR, Rastall RA (2019). Probiotics and prebiotics in intestinal health and disease: from biology to the clinic. Nat Rev Gastroenterol Hepatol.

[3] Dindo L, Lackner J (2017). Effects of different coping strategies on physical and mental health of patients with irritable bowel syndrome. Clin Gastroenterol Hepatol.

[4] Horn A, Stangl S, Parisi S (2023). Systematic review with meta-analysis: stress-management interventions for patients with irritable bowel syndrome. Stress Health.

[5] Makkawy EA, Abdulaal IE, Kalaji FR, Makkawi M, Alsindi N (2023). Prevalence, risk factors, and management of irritable bowel syndrome in Saudi Arabia: a systematic review. Cureus.

[6] (2025). Rome IV Criteria. https://theromefoundation.org/rome-iv/rome-iv-criteria/.

[7] Francis CY, Morris J, Whorwell PJ (1997). The irritable bowel severity scoring system: a simple method of monitoring irritable bowel syndrome and its progress. Aliment Pharmacol Ther.

[8] Cohen S, Kamarck T, Mermelstein R (1983). A global measure of perceived stress. J Health Soc Behav.

[9] Almansour O (2024). Prevalence of irritable bowel syndrome (IBS) in the Arab World: a systematic review. Cureus.

[10] Lovell RM, Ford AC (2012). Effect of gender on prevalence of irritable bowel syndrome in the community: systematic review and meta-analysis. Am J Gastroenterol.

[11] Sirri L, Grandi S, Tossani E (2017). Smoking in irritable bowel syndrome: a systematic review. J Dual Diagn.

[12] Lee C, Doo E, Choi JM (2017). The increased level of depression and anxiety in irritable bowel syndrome patients compared with healthy controls: systematic review and meta-analysis. J Neurogastroenterol Motil.

[13] Qin HY, Cheng CW, Tang XD, Bian ZX (2014). Impact of psychological stress on irritable bowel syndrome. World J Gastroenterol.

[14] Bennett EJ, Tennant CC, Piesse C, Badcock CA, Kellow JE (1998). Level of chronic life stress predicts clinical outcome in irritable bowel syndrome. Gut.

[15] Slouha E, Patel B, Mohamed A, Razeq Z, Clunes LA, Kollias TF (2023). Psychotherapy for irritable bowel syndrome: a systematic review. Cureus.

[16] Lackner JM, Jaccard J, Keefer L (2018). Improvement in gastrointestinal symptoms after cognitive behavior therapy for refractory irritable bowel syndrome. Gastroenterology.

[17] Altayar O, Sharma V, Prokop LJ, Sood A, Murad MH (2015). Psychological therapies in patients with irritable bowel syndrome: a systematic review and meta-analysis of randomized controlled trials. Gastroenterol Res Pract.

[18] Zernicke KA, Campbell TS, Blustein PK, Fung TS, Johnson JA, Bacon SL, Carlson LE (2013). Mindfulness-based stress reduction for the treatment of irritable bowel syndrome symptoms: a randomized wait-list controlled trial. Int J Behav Med.

